# Performance Pressure and Employee Presenteeism: The Joint Effects of Authoritarian Leadership and Independent Self-Construal

**DOI:** 10.3390/bs14030236

**Published:** 2024-03-14

**Authors:** Siyi Zhang, Haijia Wang, Qi He

**Affiliations:** International Business School, Hainan University, Haikou 570228, China; 21211202020026@hainanu.edu.cn (S.Z.); 21211202020013@hainanu.edu.cn (H.W.)

**Keywords:** performance pressure, presenteeism, authoritarian leadership, independent self-construal, self-determination theory

## Abstract

Due to the increasing competition in the market and the limited availability of high-quality employment opportunities, an increasing number of employees struggle to maintain a balance between their physical conditions and performance demands, resulting in a more widespread occurrence of “working while ill”. However, little is known about the controlled motivation behind the phenomenon under pressure. Drawing on self-determination theory, this study utilized 281 questionnaire data to examine the positive effect of performance pressure on employee presenteeism, and to explore the moderating role of authoritarian leadership and its joint moderation function effect with independent self-construal. The results indicated that performance pressure had a significant positive effect on employee presenteeism. Authoritarian leadership imposed an enhanced moderating effect between performance pressure and employee presenteeism, while independent self-construal diminished the augmentative moderating role played by authoritarian leadership in the relationship between performance pressure and employee presenteeism. This study reveals the controlled motivation of employee presenteeism under performance pressure, taking into account the cultural background and organizational context of China. Moreover, it also offers novel perspectives for effectively managing this phenomenon.

## 1. Introduction

In light of fierce market competition and drastic changes in the social environment, an escalating number of organizations are facing substantial pressure to survive. To maintain a competitive advantage, certain organizations employ cost reduction strategies such as workforce downsizing, restructuring, and the other means. And leaders convert external pressures of the organization into internal pressures of the employees to improve productivity by adjusting performance goals, raising performance demands, and adding workloads [1]. Consequently, this not only undermines employees’ work security but also imposes increased workloads upon them. Against this backdrop, the compounding pressures of work instability and excessive workloads have fostered “working while sick”, a pervasive but easy-to-ignore phenomenon [2]. This phenomenon is known as “presenteeism”, which refers to individuals continuing to working in the state of ill-health [3,4]. It has been demonstrated that presenteeism can temporarily sustain part of the performance goals and completing work tasks on time. However, it can have negative long-term effects, such as burnout [5,6] and impaired physical and mental health [7,8], as well as negative outcomes such as absenteeism and turnover intentions [9,10], even affecting organizational performance [11]. Therefore, it is important to thoroughly investigate the causes of employee presenteeism, in order to mitigate its negative impact on the sustainable development of both employees and organizations.

Existing research suggests that work stress plays a crucial role in influencing presenteeism [12]. And some studies have explored the pathways of stressors in the workplace on presenteeism based on a resource-based perspective [5,13,14]. That is, when subjected to work-related stress, employees in poor health may perceive themselves as facing potential or actual loss of resources, thereby forcing themselves to invest resources to continue working in order to maintain performance and prevent further resource loss [15]. However, there are still gaps in research when exploring the relationship between work stress and presenteeism. The literature tends to view presenteeism as a passive and reactive response of employees under stress, lacking an in-depth understanding of the proactive and adaptive nature of this behavior. As research on presenteeism deepens, Karanika-Murray and Biron [16] have pointed out that presenteeism is approach-oriented, representing employees’ active adaptation to work in order to balance performance demands despite impaired health conditions. Some scholars further divide the drivers of presenteeism into approach and avoidance motives (constructive and obsessive motives); that is, the individual may choose to work while sick because he hopes to overcome his discomfort and demonstrate loyalty to their organization. Or it may be used as a defensive strategy to avoid tarnished image or receiving punishment [15,17,18]. Additionally, performance pressure, as opposed to general work pressure, refers to the organization’s demand for high performance from its employees. This expectation of high performance can be intimidating to the employee, but it can also increase their focus on the benefits associated with achieving performance goals, leading to the willingness and motivation to prioritize current performance demands [19]. Therefore, performance pressure creates a sense of threat and goal orientation, motivating employees to cope with demands and achieve equilibrium. This aligns with the characteristic of presenteeism, which seeks benefits and avoids harm, and also corresponds to the motivation paradigm in self-determination theory. Given this, this study aims to incorporate self-determination theory to elucidate presenteeism, and attempts to view presenteeism as a proactive work practice undertaken by individuals in the context of performance pressure. 

In addition, certain scholars have called for exploring the boundaries within which employees make “trade-offs” between performance goals and health [16]. According to self-determination theory, environmental factors and individual characteristics can influence activation of different types of motivation, thereby impacting corresponding behavioral outcomes under different motivational types [20,21]. Therefore, this study proposes the integration of organizational situational factors (authoritarian leadership) and individual factors (independent self-construal) to build a triple interaction model, in an attempt to comprehensively explain the conditions for the occurrence of presenteeism under the context of performance pressure. Whether an individual chooses presenteeism is influenced by a combination of factors from both organizational and individual characteristics. Leadership behavior, as an important aspect of organizational situational factors, plays a significant role in influencing employee behavior. Empirical studies have demonstrated that leadership styles that endorse health and supportive leadership behaviors effectively minimize presenteeism [22,23]. On the other hand, when leaders are distrusted and attendance pressure increases, the likelihood of presenteeism rises [6,10]. Thus, it is evident that leaders can affect employees’ thinking, attitudes, and actions by implementing additional control measures. As a local Chinese style of leadership behavior, authoritarian leadership emphasizes the leader’s personal authority and strict dominance over subordinates [24]. However, previous research mainly focuses on the effects of authoritarian leadership on presenteeism among healthcare professionals [25], with certain limitations in the sample used. Thus, this study supposes that in high-pressure performance situations, the authoritarian leadership style of supervisors may affect employees’ perception of performance goals, increasing their external motivation and level of internalization, thereby intensifying controlled motivation for presenteeism. Currently, when employees are in poor health, presenteeism is often viewed as a means of coping with strict leadership control and meeting performance targets. 

Self-determination theory suggests that employees with different traits will have different levels of internalization in response to leader influence [26]. Authoritarian leadership can lead to varying degrees of external stress in subordinate interactions, yet perceptions of this external stress can be moderated by individual trait factors. And individuals with high levels of independent self-construal have a strong sense of independence and prioritize the expression of intrinsic traits. They often adopt behaviors that are consistent with their own traits and perceptions [27,28]. Furthermore, existing studies have shown that personality factors such as independent self-construal play an important moderating role in the relationship between leadership style and employee behavior [29,30]. Therefore, this study hypothesizes that employees with high levels of independent self-construal will experience limited psychological and changes when faced with authoritarian leadership, and will be more likely to perceive their behavior as autonomous. When deciding whether to engage in presenteeism under pressure to perform, these employees are less likely to take into account possible criticism and rejection from their leaders. This reduces the excessive control exerted by authoritarian leaders over employees and undermines the positive moderating effect of authoritarian leadership on both performance pressure and presenteeism.

In summary, this study aims to examine the situational factors and boundary conditions of presenteeism among employees through the lens of self-determination theory. This research seeks to advance the current understanding in three key areas: Firstly, this study aims to account for the motivation for presenteeism by analyzing the controlled motivation within self-determination theory. Specifically, this study examines how performance pressure influences employees’ decision to work while ill and contributes to understanding presenteeism motivation. Secondly, the current study develops a multiple moderation model of authoritarian leadership and independent self-construal, further revealing the boundary conditions under which performance pressure affects employees’ presenteeism. That is, authoritarian leadership amplifies the controlled motivation and raises the tendency of employee presenteeism in stressful situations. And the tendency depends, to a certain extent, on the level of independent self-construal. Finally, this study can expand to some extent the research on the mechanisms of presenteeism in organizational contexts, and provide some insights for organizations to manage presenteeism. 

## 2. Theoretical Background and Hypothesis Development

### 2.1. Presenteeism and Self-Determination Theory

Presenteeism, first introduced by Cary Cooper [31], describes the behavior of individuals who continue to attend work even when they feel unwell or uncomfortable in the working environment for extended periods. Further studies on presenteeism have led to divergent definitions. Certain scholars consider it as reduced productivity caused by personal health problems, thereby underscoring its potential economic impact on organizations [32,33,34]. As a phenomenon, presenteeism has recently gained more academic focus [35], whereas most scholars have defined presenteeism as working while ill [3,17,36], and have developed the term “sickness presenteeism”. This definition is more focused on the behavior itself, aiming to understand the antecedents and consequences of presenteeism, as well as the motivations behind this behavior, and has therefore been widely used in subsequent empirical research [15,35]. Ruhle et al. [4] suggest that this behavior should be understood more broadly and not be limited to the illness itself, suggesting that presenteeism be understood as behavior of working in the state of ill-health to cover all types of health conditions and can be applied to any type of work. Thus, this study employs Ruhle et al.’s [4] definition of presenteeism to enhance comprehension and investigate the underlying mechanisms.

Currently, prior studies have elucidated the mechanisms of presenteeism from a behavioral motivation perspective [37,38]. For example, Lu, Lin, and Cooper [39] suggest that presenteeism is driven by two factors: approach motivation and avoidance motivation. These aspects reveal individuals’ behavior traits of pursuing advantages and evading harm when confronted with pressure [15]. As research progresses, scholars have expanded their comprehension of the driving forces of presenteeism by incorporating self-determination theory [16,18]. It has been suggested that behavioral motivation can be classified under two categories: autonomous motivation and controlled motivation. The former emphasizes self-actualization, personal values, and self-esteem, while the latter places a high value on salary and other external rewards, such as praise, rewards, and security. Employees with controlled motivation tend to adhere to “external” or “must” mandatory behavioral standards when choosing presenteeism.

Therefore, this study introduces self-determination theory to better understand and explore employee presenteeism in the context of performance pressure. Self-determination theory emphasizes that individuals have an active role in the motivation process and make behavioral choices based on a thorough comprehension of their intrinsic requirements and external environmental information [40]. Performance pressure, as a specific type of work pressure, involves high performance expectations for employees. This can motivate employees to focus on the benefits associated with performance outcomes, generating the willingness, motivation, and behavior to achieve current performance goals [19], which can lead to employee presenteeism. It can be seen that the independent variable (performance pressure) and the dependent variable (presenteeism) investigated in this study are both related to employees’ individual work motivation. Thus, this study believes that self-determination theory aligns well with the research framework and provides an appropriate theoretical basis for understanding the motivation for presenteeism. 

### 2.2. Performance Pressure and Employee Presenteeism

Performance pressure is commonly considered a stressor among scholars, symbolizing the high performance expectations set by organizations or the external environment [19,41,42]. Accomplishing performance needs can bring about various benefits for employees, including salary increases, career advancement, and personal development. Conversely, failure to meet these standards can have negative consequences for employees [1]. Performance pressure can cause employees to feel a sense of urgency and tension in achieving their goals and avoiding negative consequences [19]. This can lead to cognitive and emotional changes that affect their behavior [43]. According to self-determination theory (SDT), individual behavior is driven by different motivations [44]. Motivations are divided into autonomous motivation and controlled motivation depending on the level of autonomy. Autonomous motivation manifests as a strong inclination towards behavior based on the individual’s full identification with the intrinsic value of the behavior [21,45]. In contrast, controlled motivation is the inclination towards engaging in behavior based on the external stimuli that the behavior brings [21,45]. When employees encounter stressful external environments, they will evaluate the situation, generating controlled motives [40]. In such instances, their behavioral intentions are stable and influenced by external regulations and internalization [21,45]. Given this, this study posits that performance pressure is both threat- and goal-oriented, ultimately leading to enhanced controlled motivation among employees, and actively encouraging presenteeism. 

On one hand, performance pressure exerted on employees is viewed as external pressure, which can create a sense of threat and a mismatch between their current performance and the organizational performance objectives [1]. This implies that, in exchange for future financial rewards or to avoid punitive action, employees must take action to improve their performance [19]. In certain situations, employees may experience anxiety about potential losses and feel pressure to conform, leading to a desire for safety and incentives. This can result in an increase in controlled motivation for employee presenteeism, where employees prioritize external work incentives over their own physical comfort and autonomy. This behavior is driven by the desire to preserve specific rewards [46] and punishment or other detriments [17]. On the other hand, performance pressure, partly reflecting the organization’s focus on achieving superior performance outcomes [19,46,47], can increase employees’ awareness of the need to achieve performance goals and high-quality outcomes [48]. This can lead them to adopt introjected regulation—a more internalized form of external regulation that still emphasizes external control. Employees will strive to achieve performance goals in order to increase their self-esteem and sense of worth by following the “must” criteria [49]. Thus, the goal-oriented nature of performance pressure causes employees to experience tension and anxiety, leading them to internalize exterior stimuli and regulations, and intensifying their controlled motivation. Even if they suffer from illness or ill-health, employees willingly attend work to meet the organization’s performance expectations by achieving performance goals. Thus, the following hypothesis is proposed:

**Hypothesis 1 (H1).**  *Performance pressure exerts a significant positive effect on employee presenteeism*.

### 2.3. The Moderating Role of Authoritarian Leadership

Leadership style has a significant impact on the attitudes and behaviors of employees [50]. Authoritarian leadership is defined by leaders who emphasize personal authority and exert strict control over subordinates, expecting absolute compliance [24]. Essentially, authoritarian leadership reflects the unequal relationship between leaders and subordinates in traditional Chinese organizations, where leaders establish authority via autocratic practices, belittlement, image-building, and instructive behaviors. Employees typically exhibit compliance, submissiveness, reverence, and shame in response to certain stimuli [24]. Extensive research has shown that authoritarian leadership has a detrimental effect on job satisfaction, performance, and well-being in the workplace. This can result in increased pressure and negative emotions among employees [51]. According to self-determination theory, authoritarian leadership influences employee behavioral motivation. Employees under authoritarian leadership may feel that they have less control over their behavior, particularly if they are expected to perform tasks that align with the leader’s expectations. This can reduce their sense of self-determination and increase their sense of external control [44].

This study argues that authoritarian leadership increases the likelihood of employee presenteeism in performance pressure scenarios. Firstly, authoritarian leaders maintain tight control over their employees, emphasizing strict hierarchical relationships. Their autocratic practices do not allow employees to ask questions or make suggestions, but rather assigns tasks and enforces rules. This behavior can deprive employees of their sense of control and autonomy over their work [52]. It can prevent them from autonomously controlling the process and outcome of their work [53], and thus, they have to opt for presenteeism to maintain work progress. Furthermore, authoritarian leaders often disregard the contributions and suggestions of their employees, undervalue and suppress their subordinates’ abilities, strictly control information, and prioritize their own authoritative image [54]. This belittling and image-building behavior increases the social and psychological distance between employees and leaders [55], reducing communication and interaction between the two parties; at the same time, it can also make employees fearful of expressing their willingness to take a break from work and make adjustments [56,57]. Finally, authoritarian leaders set high performance standards for employees and are excessively concerned about their performance. They may even reprimand under-performing employees to their faces, showing instructive reprimanding behaviors that imply higher performance expectations. This can cause employees to develop mentalities such as anxiety and panic [58], and amplify the desire for extrinsic rewards and security. In this situation, employees strive to improve their performance to avoid punishment for incomplete tasks, while also increasing the likelihood of presenteeism to receive recognition and rewards from their superiors. In summary, autocratic practices, belittlement, image-building, and reprimanding styles from authoritarian leadership enhance the controlled motivation for presenteeism in performance pressure situations, where employees are more likely to view presenteeism as a stress response strategy [59]. Thus, the following hypothesis is proposed:

**Hypothesis 2 (H2).**  *Authoritarian leadership plays an enhancing moderating role in the relationship between performance pressure and employee presenteeism*.

### 2.4. The Moderated Moderation Effect of Independent Self-Construal

According to Markus and Kitayama [27], self-construal refers to an individual’s tendency to choose a reference system for self-perception. This choice can affect the individual’s perception of changes in relationships, which in turn affects individual’s behavioral tendencies. Self-construal includes both independent self-construal and dependent self-construal. Individuals with independent self-construal tend to express their self-concept through the characteristics that distinguish them from others, emphasizing the uniqueness and independence of their behavior and attitudes [28]. Those with a high level of independent self-construal tend to focus on personal goals and achievements, pursuing individual autonomy and self-worth in their behavior. Their values and behaviors are less influenced by others [27], which makes them less concerned about the opinions of leaders and colleagues in their work [60]. Therefore, when employees face the same or similar leadership styles, individuals with different levels of independent self-construal may perceive leadership behaviors differently, leading to different coping mechanisms [27]. For example, it may mitigate the negative impact of abusive management on employees’ psychological contract breach [30]. 

Following self-determination theory, there are differences in causal orientation between individuals, and these differences can affect their perception of their own behavioral intentions. Specifically, individuals with an autonomous orientation are more likely to perceive their behavior as autonomous, while individuals with a controlled orientation are more likely to perceive their behavior as externally controlled [21,44]. Thus, this study proposes that individuals with high independent self-construal are prone to perceive their behaviors as autonomous and have a higher level of autonomous orientation. This perception would diminish the sense of extrinsic control that authoritarian leadership imposes on the individual, thereby interfering with the augmentative regulation of authoritarian leadership. On the one hand, employees who have high independent self-construal tend to be self-centered. These employees have a stronger sense of individual subjective well-being and stronger psychological capital [61,62], and they also tend to maintain a greater interpersonal distance from their leaders and are less sensitive to their leaders’ attitudes and behaviors. Thus, in confronting authoritarian leadership from their superiors, employees are less likely to overthink the leadership factor and worry about the leader’s perception of their behavior. As a result, this mitigates the impact of authoritarian leadership on them. Conversely, employees with low independent self-construal are more inclined towards fostering good interpersonal relationships [63]. They tend to be overly considerate of others’ feelings and are more sensitive to the attitudes and behaviors of their leaders. Consequently, these employees will feel the pressure and demands from the leader more strongly when faced with an authoritative leader. They will be more inclined to view presenteeism as a means of coping and reducing performance pressure.

On the other hand, individuals with high independent self-construal exhibit greater autonomy [64,65]. They act out of their own volition and fully identify with the value and significance of their behavior [27]. Therefore, employees with high independent self-construal act based on their “true” selves in various contexts prioritize the expression of their inner selves, and prove themselves through their actions [28]. When faced with authoritarian leadership, these employees perceive behavior as autonomous, and their psychological and behavioral changes and influences are relatively minor. When it comes to the question of whether or not to work while ill-health, employees often make choices based on their personal needs rather than meeting the leader’s expectations. In contrast, employees with low independent self-construal place more emphasis on relationships with others and are more susceptible to external influences. They tend to be more loyal to their roles in the organization, and value the thoughts and opinions of their leaders, colleagues, and the group [27]. Thus, employees with low independent self-construal tend to exhibit subservient behavior that are subservient to the leader’s expectations to avoid being blamed, and have a stronger controlled motivation for presenteeism, thereby increasing the likelihood of presenteeism. Thus, the following hypothesis is proposed:

**Hypothesis 3 (H3).**  *Independent self-construal weakens the enhancing moderating effect of authoritarian leadership on performance pressure and employee presenteeism*.

**Figure 1:**
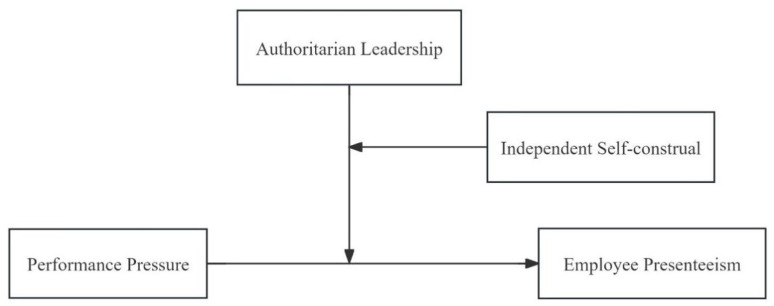
Theoretical model.

The theoretical model of this study is shown in Figure 1.

## 3. Materials and Methods

### 3.1. Sample and Procedure

Data were collected by our research team through an online survey distributed via the *Credamo* platform from 23 April to 10 May 2023. This survey targets employees of Chinese enterprises, including those in logistics, manufacturing, tourism services, banking, and sales industries. The participants include both general staff (such as administrative, technical, and business function employees) and service staff (such as sales personnel). The participants were selected randomly by their respective enterprises. To ensure the authenticity, validity, and reliability of the data, several control measures were implemented. A limit of one entry per IP was set for the questionnaire, and an attention test was set in the formal questionnaire to check the validity of the questionnaire. A total of 330 questionnaires were distributed, and after excluding invalid samples such as too short answers, answering the same data consecutively, and failing the attention test, 281 valid questionnaires were retained. Among them, there were 43 questionnaires from logistics companies, 63 from manufacturing companies, 56 from tourism service companies, 54 from banking companies, and 65 from sales companies, indicating a relatively even distribution. The effective response rate of the questionnaire was 85.15%. 

Among the 281 valid samples collected, male employees accounted for 41.6% and female employees accounted for 58.4%, indicating a relatively balanced gender ratio. In terms of age, the majority of participants were young and middle-aged employees between 25 and 41 years old. Regarding educational level, 6.1% had a college diploma or below, 77.9% had a bachelor’s degree, and 15.3% had a master’s degree or above, indicating a higher proportion of knowledge-based employees. Concerning the current organizational type, 1.8% were from national government agencies, 20.3% were from state-owned enterprises, 67.9% were from private enterprises, and 10% were from foreign-funded and other organizations. In terms of years of work time, 1.8% had less than 1 year of experience, 33.8% had 1–5 years, 42.3% had 6–10 years, and 22.1% had more than 10 years, indicating a generally high level of employee stability. The general characteristics pf the valid samples are as shown in Table 1.

### 3.2. Measures

The variables to be measured in this study include performance pressure, employee presenteeism, authoritarian leadership, independent self-construal, and control variables. All scales were selected from validated mature scales both domestically and internationally. The translation–back translation method was used to convert the English scales and make appropriate revisions to ensure the appropriateness of their wording in a Chinese context [66]. Unless otherwise specified, the employees were required to report each item on a 5-point Likert scale from 1 (strongly disagree) to 5 (strongly agree). The specific survey items are presented in Appendix A.

Performance pressure: The scale developed by Mitchell et al. [42] was used and validated with a Chinese sample by Li et al. [67]. It consists of 4 items, such as “The characteristic of my workplace is performance-oriented”. The Cronbach’s α in this study is 0.875.

Authoritarian leadership: The scale of authoritarian leadership used in the study by Zheng et al. [68] has been empirically validated in a Chinese context [55]. It consists of 5 items, such as “My leader determined all decisions in the organization whether they are important or not”. The Cronbach’s α in this study is 0.9.

Independent self-construal: Based on the self-construal scale developed by Singelis [28] to directly measure self-construal, and in combination with the discussion of the applicability of this scale in China by the domestic scholars Pan and Lv [69], we ultimately chose to use six items of the independent self-construal dimension from the scale developed by Singelis. Sample items include “My personal identity independent of others, is very important to me”. In this study, the Cronbach’s α is 0.775.

Employee presenteeism: Drawing on the measurement methods of presenteeism by Aronsson et al. [3] and Lu et al. [17], translated by domestic scholars such as Sun and Zhang [15]. This measurement method includes 2 items, asking respondents to “Recall whether you have experienced the following situations in the past 6 months: 1. Although you feel sick, you still force yourself to go to work. 2. Although you have physical symptoms such as headache or backache, you still force yourself to go to work”. In this study, the Cronbach’s α is 0.9.

Control variables: Referring to the literature on presenteeism, this study selected the following control variables: gender, age, education level, years of work experience, and organizational type.

## 4. Data Analysis and Results

This study firstly conducted Common Method Bias tests using SPSS 26.0, followed by Confirmatory Factor Analysis (CFA) using Amos 24.0. Descriptive statistics, correlation analysis, and hierarchical regression analysis were then performed on the variables using SPSS 26.0. Additionally, the simple slope test for moderated effects was conducted using the BOOTSTRAP method in the PROCESS macro.

### 4.1. Common Method Bias

Since the questionnaire data are all from self-assessment reports of employees, in order to avoid the influence of common method bias on the research results, Harman’s single-factor test was used to test for common method bias. The results showed that after conducting a non-rotating factor analysis, there were 4 factors with eigenvalues greater than 1, which is consistent with the dimensional division of this study. The eigenvalue of the first factor was 5.006, and the percentage of variance explained was 29.448%, which is less than the recommended value of 40%. Therefore, it can be considered that the common method bias of the data in this study is within an acceptable range.

### 4.2. Confirmatory Factor Analysis

This study performed confirmatory factor analysis (CFA) using Amos 24.0 to test the factor model. It is necessary to examine the discriminant validity of performance pressure, authoritarian leadership, independent self-construal, and employee presenteeism. The results (see Table 2) indicated that the hypothesized four-factor model had a better fit (χ^2^ = 183.454; df = 113; χ^2^/df = 1.623; RMSEA = 0.047; SRMR = 0.056; CFI = 0.969; TLI = 0.962) than other alternative models. Therefore, it can be seen that there is good discriminant validity among the latent variables in this research model.

### 4.3. Descriptive Statistics and Correlation Analysis Results

The means, standard deviations, and correlation coefficients of the main variables are shown in Table 3. Performance pressure and employee presenteeism showed a significant positive correlation (r = 0.501, *p* < 0.001). Authoritarian leadership was possibly associated with employee presenteeism (r = 0.350, *p* < 0.001), while independent self-construal and employee presenteeism showed a significant negative correlation (r = −0.117, *p* < 0.05), providing preliminary evidence for hypothesis testing in subsequent analyses.

### 4.4. Hypothesis Testing Analysis

To test the hypotheses of the direct effect, two-dimensional interaction, and three-dimensional interaction, this study employed hierarchical regression analysis [70]. The main effects of performance pressure on presenteeism, as well as the moderating effects of authoritarian leadership and independent self-construal, were examined in sequence.

#### 4.4.1. Testing the Impact of Performance Pressure on Employee Presenteeism

H1 assumes that performance pressure has a positive impact on presenteeism. To test H1, this study adopted the hierarchical regression analysis, and the results are shown in Table 4. Model 2 significantly predicted presenteeism (F = 17.136, *p* < 0.001), with a 25% increase in the amount of variance explained by the model. This suggests that performance pressure explained 25% of the variance in presenteeism and was significantly positively correlated with employee presenteeism (β = 0.563, *p* < 0.001, Model 2). Therefore, H1 was supported.

#### 4.4.2. Testing the Moderating Effect of Authoritarian Leadership

H2 assumes that authoritarian leadership plays an enhancing moderating role in the relationship between performance pressure and presenteeism. First, based on Model 2, authoritarian leadership was introduced to construct Model 3. Then, on the basis of Model 3, the interaction term between centered performance pressure and authoritarian leadership was introduced to construct Model 4, thus examining the moderating effect of authoritarian leadership.

**Figure 2:**
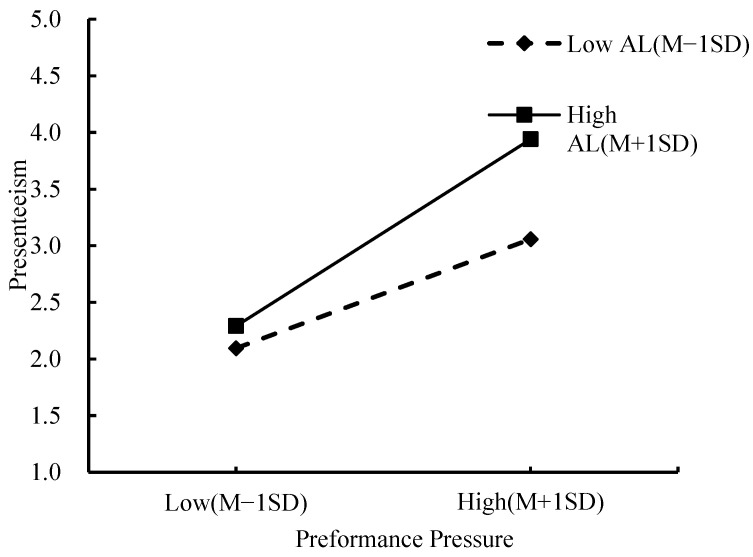
The moderating role of authoritarian leadership.

The results (shown in Table 4) indicated that the interaction between performance pressure and authoritarian leadership had a significant positive impact on presenteeism (β = 0.114, *p* < 0.05, Model 4) and was consistent with the regression effect of performance pressure on presenteeism (β = 0.504, *p* < 0.001, Model 4). Thus, the results provided initial support for H2. Additionally, this study used PROCESS macro to test H2, and adopted [71] procedures to plot the interaction effect. As shown in Table 5 and Figure 2, under low authoritarian leadership conditions (Mean − 1 SD), the effect of performance pressure on employee presenteeism was significant (Effect = 0.385, *p* < 0.001). Under high authoritarian leadership conditions (Mean + 1 SD), this relationship remained significant and was significantly enhanced (Effect = 0.660, *p* < 0.001). Therefore, H2 was supported.

#### 4.4.3. Testing the Second-Order Moderating Effect of Independent Self-Construal

H3 suggests that independent self-construal attenuates the enhancing effect of authoritarian leadership on performance pressure and employee presenteeism. To better test this effect, we used SPSS 26.0 for multiple linear regression. Independent self-construal was introduced to form Model 5 on the basis of Model 4. Then, based on Model 5, the interaction term of centered performance pressure, authoritarian leadership, and independent self-construal is introduced to construct Model 6. Based on Model 6, we constructed Model 7 by introducing the other two two-way interaction terms. The results (shown in Table 4) showed that the regression effect of the three-dimensional interaction terms of performance pressure, authoritarian leadership, and independent self-construal on employee presenteeism was significantly negative (β = −0.248, *p* < 0.01, Model 6), and the regression effect of the two-dimensional interaction of performance pressure and authoritarian leadership on employee presenteeism was in the opposite direction (β = 0.116, *p* < 0.05, Model 6). After adding all the interaction terms, the results of Model 7 show that the three-way interaction still has a significant statistical significance on employee presenteeism (β = −0.186, *p* < 0.05, Model 7), which proves that independent self-construal can weaken the moderating effect of authoritarian leadership on the relationship between performance pressure and employee presenteeism. H3 was initially supported.

In addition, this study conducted a simple slope test for the three-way interaction moderation using PROCESS macro (shown in Table 6). The results were consistent with the regression results. The results were consistent with the regression results. Presenteeism under the two combinations of high authoritarian leadership (High AL–Low ISC: Effect = 0.7146, *p* < 0.001; High AL–High ISC: Effect = 0.5334, *p* < 0.001) were both higher than presenteeism under the combination of low authoritarian leadership (Low AL–High ISC: Effect = 0.5067, *p* < 0.001; Low AL–Low ISC: Effect = 0.1674, *p* = 0.1377, ns). It indicates that authoritarian leadership positively moderates the relationship between performance pressure and employee presenteeism. However, presenteeism under the combination of high authoritarian leadership and high independent self-construal (Effect = 0.5334, *p* < 0.001) was significantly lower than presenteeism under the combination of high authoritarian leadership and low independent self-construal (Effect = 0.7146, *p* < 0.001). This further supports the three-way interaction of H3.

**Figure 3:**
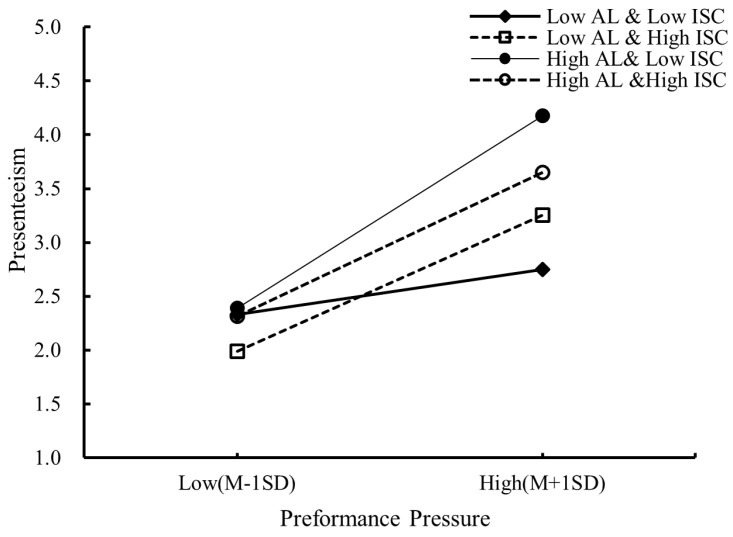
The moderating role of independent self-construal.

Finally, this study plotted a simple slope figure of the three-way interaction (shown in Figure 3). The overall slope of the effect of performance pressure on employee presenteeism was positive. Figure 3 shows that under high authoritarian leadership, the overall slope of presenteeism for employees with high independent self-construal was flatter than that for employees with low independent self-construal. It further illustrates that independent self-construal played a negative moderating role; that is, the higher the level of independent self-construal, the weaker the moderating effect of authoritarian leadership on the relationship between performance pressure and employee presenteeism. Thus, H3 was graphically supported here.

## 5. Conclusions and Discussion

This study, grounded in self-determination theory, examines the relationships between performance pressure, authoritarian leadership, independent self-construal, and their impact on employee presenteeism by constructing a model, collecting data, and conducting empirical tests. Table 7 presents the hypotheses and a summary of the test results.

Firstly, this study confirms a significant positive impact of performance pressure on employee presenteeism, thereby supporting Hypothesis 1. The research indicates that the combination of the threat and goal-oriented nature of performance pressure stimulates a controlled motivation for presenteeism. In situations of performance pressure, they will be more concerned about the potential risks and economic benefits associated with inadequate performance, which leads them to opt for presenteeism. This finding aligns with the previous literature where continuous and intermittent stressors in the workplace are crucial factors influencing older employees’ decisions to work while ill [14]. Additionally, high job demands and workload have been shown to promote presenteeism [5]. Therefore, performance pressure, high job demands, and workload in the workplace are significant factors contributing to employee presenteeism. These factors increase employees’ workload and elevate their stress levels, often leading employees to continue working even when their health is compromised. While working while ill temporarily may sustain a certain level of performance, prolonged high-pressure environments and presenteeism can further deteriorate employees’ physical and mental health [5], creating a detrimental cycle. Hence, creating a healthy and efficient work environment is crucial for reducing presenteeism among employees and fostering mutual growth for both the organization and individuals.

Secondly, this study confirms the augmentative moderating effect of authoritarian leadership in the relationship between performance pressure and employee presenteeism, whereas independent self-construal weakens the moderating effect of authoritarian leadership on performance pressure and employee presenteeism, thus supporting Hypothesis 2 and Hypothesis 3. Previous research has shown that authoritative leadership has a positive effect on nurse presenteeism [25], which is consistent with the findings of this study. However, these studies failed to explore the relationship between leadership behavior and subordinate perception consistency, ignoring the comprehensive consideration of the effects of the interaction between individual traits and leadership behaviors on employee presenteeism. And it has been found that independent self-construal can mitigate the extent to which negative leadership styles influence employees’ maladaptive behaviors [30]. In response to recent literature calls, this study examines the interaction between authoritarian leadership and independent self-construal on employee presenteeism in the context of performance pressure from the perspective of self-determination theory. It finds that authoritarian leadership plays an enhancing moderating role in the relationship between performance pressure and employee presenteeism. Authoritarian leaders, through autocratic practices, belittlement, image-building, and instructive behaviors, further strengthen employees’ motivation to attend work, which increases the likelihood of employees choosing presenteeism under performance pressure. However, individual trait factors moderated the perceived pressure from authoritative leaders. Employees with high independent self-construal, compared to those with low levels, have a stronger sense of self-orientation and perceive more autonomy in their behavioral intentions. This, to some extent, limits the enhancing effect of authoritarian leadership on the relationship between performance pressure and employee presenteeism.

### 5.1. Theoretical Implications

First, to some extent, it expands the research on the outcome variables related to performance pressure. Most existing studies have investigated performance pressure as a stressor and antecedent variable, exploring the impact of performance pressure on employee attitudes and behaviors. For example, moderate performance pressure helps promote employee work engagement [72] and improve employee performance [41]. Simultaneously, some studies have pointed out that performance pressure may bring cognitive conflict to employees, exacerbate their state of ego depletion, and lead to workplace anxiety [67] and emotional exhaustion [42]. However, these studies rarely focus on the impact of performance pressure, a specific stressful situation, on employee presenteeism. Therefore, this study confirms the positive effects of performance pressure on employee presenteeism based on self-determination theory. It also highlights that performance pressure has both threatening and goal-oriented qualities, which can promote the occurrence of employee presenteeism. This expands the research on the subsequent outcomes of performance pressure on employee presenteeism.

Furthermore, it uncovers the controlled motives behind employee presenteeism in stressful situations. Previous studies on pressure and presenteeism, while also emphasizing the external sense of control of stress, have only argued for negative employee reactions [5,13,14], ignoring the goal-oriented nature of employee presenteeism. Drawing from the controlled motivation perspective of self-determination theory, this study examines the impact of performance pressure on employee presenteeism and proposes that physically unfit employees internalize the external stimulus of pressure to some extent as a perceived necessity to achieve their goals. They will view presenteeism as a proactive coping strategy to achieve performance goals, obtain rewards, and avoid potential risks, thereby reducing inner discomfort and maintaining self-esteem and a sense of accomplishment. Therefore, this study considers presenteeism as an adaptive behavior employed by employees to balance physical limitations and performance demands under pressure, which theoretically deepened the functional cognition of employee presenteeism. It responds to the call for a comprehensive examination of presenteeism motivation raised in previous research [18] and to some extent compensated for the fact that previous studies used resource conservation theory, a work demand resource model, and social cognitive theory to explain the limitations of presenteeism.

Finally, the boundary conditions for the relationship between performance pressure and employee presenteeism were further clarified by considering the influences of leadership style and personality traits on individual behavior. Existing studies exploring this topic have only partially investigated the mechanisms by which workplace stressors influence presenteeism. For example, persistent stressors in the workplace [14], higher job demands [5], and overemphasis on the requirement of attendance [73] can elevate employees’ stress levels, leading to their tendency to work while suffering from ill-health in order to maintain their performance levels. These studies have not examined the moderating effects of specific situational factors and personality traits on this relationship. Drawing from self-determination theory, this study introduces a higher-order moderation to uncover the various boundary conditions that influence employee presenteeism under performance pressure. Authoritarian leadership, characterized by practices such as autocratic practices, belittlement, image-building, and instructive behaviors, exerts additional control pressure on employees, thereby increasing the likelihood of employee presenteeism under performance pressure. However, the extent to which this control pressure influences presenteeism depends on the level of independent self-construal. Therefore, this study further enriches the research on presenteeism situations by exploring the differences in the choices made by different employees when faced with the same situation. It enhances the understanding and interpretation of presenteeism phenomena and provides organizations with a new theoretical perspective on managing this phenomenon.

### 5.2. Practical Implications

Firstly, enterprises need to manage performance pressure scientifically. Although some studies point out that performance pressure can promote the development of employees’ potential and improve their intrinsic motivation and creativity, with the depth of research, scholars have pointed out that excessive performance pressure can become a burden for employees, leading to their physiological and psychological overwork and even illness [42,74]. Therefore, enterprises should consciously take measures to scientifically and rationally manage employees’ performance pressure, in order to prevent the negative impact of excessive pressure on their physical and mental health. Specifically, enterprises should set clear and achievable goals for employees across different roles, and engage in consistent communication, providing immediate feedback and suggestions. This approach can help employees understand their work performance and expected objectives clearly. Moreover, it is recommended that companies improve their communication channels and encourage employees to express their views, concerns, and challenges freely. This will help prevent presenteeism caused by performance pressure. Simultaneously, it is indispensable to establish a reasonable sick leave performance system. This should include providing a certain amount of paid sick leave and simplifying the sick leave application process, thereby reducing the psychological burden on employees. Within the performance evaluation system, emphasis on working hours can be reduced in favor of greater attention to work efficiency, quality, and health management. This will encourage employees to adopt healthy work practices, such as reasonable planning of working hours and avoiding prolonged overtime work. Additionally, the rapid advancement of internet communication technology has progressively eliminated constraints on work hours and locations. Enterprises should capitalize on this trend by offering employees flexible work arrangement options, such as allowing them a degree of freedom in scheduling their work hours or the option of choosing remote work. This strategy aims to balance physical constraints and work demands, thereby reducing presenteeism triggered by performance pressure and potential health risks.

Secondly, enterprises need to effectively prevent and mitigate the negative impacts of authoritarian leadership. Although authoritarian leadership helps to improve decision-making efficiency and strengthen execution, as the new generation employees who advocate individuality, freedom and openness gradually become the main force of enterprises, they put forward new demands on leadership behavior [75]. Therefore, enterprises should focus on the effectiveness of leadership behaviors. When shaping leadership styles, it is important to minimize the adverse impacts of authoritarian leadership. Specifically, enterprises can offer leadership development training to assist managers in comprehending the impact of their leadership style on employee behavior and attitudes. This can help managers identify and reduce authoritarian leadership behaviors while learning to use multiple leadership styles effectively. Simultaneously, when managing individual employees, managers should delegate authority appropriately and empower employees to improve and adjust their work styles. This is particularly important for employees with poor health conditions. Where necessary, managers should assist these employees in adjusting their work styles or processes as needed and provide access to psychological and health counselling services to guide employees in focusing on their own health management.

Finally, enterprises should deeply understand and shape employees’ independent self-construal. Employees with high independent self-construal exhibit traits of non-attachment, independence, and a focus on self-value realization, as well as strong self-management skills [27]. To achieve this, enterprises should fully utilize organization’s resources to conduct regular questionnaire surveys and one-on-one communication to gain a deeper understanding of the personality traits, values, and goals of their employees. This approach can guide employees in identifying their strengths and growth paths, thereby effectively shaping their independent self-construal effectively. Furthermore, it is important for enterprises to emphasize the characteristic of independent self-construal in employees by enhancing their autonomy in the workplace and providing them with more opportunities, rights, and resources to work autonomously. This can help employees to demonstrate and prove their abilities. By doing this, employees can rationally assess and adjust their abilities, resources, and conditions, even when facing mental and physical health challenges. This can help to minimize presenteeism and their detrimental effects on health.

### 5.3. Limitations and Future Prospects

Firstly, all variables in this study come from employees’ self-assessments. Specifically, the measurement of presenteeism relies on employees’ self-perceived data. However, it is important to acknowledge that some participants may exaggerate their tendency towards presenteeism due to impression management and other reasons, leading to subjective cognitive biases in the research results. To enhance the scientific rigor of future studies, it is suggested to consider using methods such as experimental observation to obtain multi-source data on presenteeism.

Furthermore, this study examines the influence and boundary conditions of performance pressure on employee presenteeism from the perspective of self-determination theory. However, previous research has primarily focused on using self-determination theory to explain the formation mechanisms of individual motivation and psychological basic needs. In contrast, this study focuses on analyzing the conditions under which presenteeism occurs as a result of external factors, making it difficult to consider mediating mechanisms at the motivational and psychological level. Therefore, future research can explore potential mediating mechanisms to comprehensively understand how performance pressure affects employee presenteeism.

Finally, this study only explores the influence of performance pressure on employee presenteeism based on self-determination theory, without further investigating the subsequent outcomes of this behavior. Existing research has confirmed the negative impact of presenteeism, and some scholars have also begun to explore the potential positive effects of presenteeism, such as higher performance evaluations and job redesign-related behaviors [76,77]. However, it is evident that there is no consensus regarding the subsequent outcomes of presenteeism. Thus, future research can extend the current theoretical model to explore the mechanism of action of positive or negative outcomes that may be triggered by presenteeism and to refine the perception of presenteeism.

## Figures and Tables

**Table 1:** General characteristics of the sample.

Sortation		Frequency (Number)	Percentage (%)
Sex	Man	117	41.6
	Woman	164	58.4
Age	<25	14	5
	25–30	107	38
	31–40	137	48.7
	41–50	21	7.5
	>50	2	0.8
Education	High school and below	2	0.7
	Associate degree	17	6.1
	Bachelor’s degree	219	77.9
	Master’s degree and above	43	15.3
Work Time/Year(s)	<1	5	1.8
	1–5	95	33.8
	6–10	119	42.3
	>10	62	22.1
Organization Type	National Government Agencies/Institutions	5	1.8
	State-owned enterprises	57	20.3
	Private enterprise	191	67.9
	Foreign enterprise	27	9.6
	Other	1	0.4

Note: N = 281.

**Table 2:** Confirmatory factor analysis.

Model	χ^2^	df	χ^2^/df	GFI	TLI	CFI	RMSEA	SRMR
Four-Factor Model (PP, AL, ISC, EP)	183.454	113	1.623	0.928	0.962	0.969	0.047	0.056
Three-Factor Model (PP, AL + ISC, EP)	539.331	116	4.649	0.766	0.780	0.812	0.114	0.113
Two-Factor Model (PP + AL + SC, EP)	1099.368	118	9.317	0.607	0.499	0.565	0.172	0.218
Single-factor model (PP + AL + ISC + EP)	1361.053	119	11.437	0.562	0.371	0.450	0.193	0.227

Note: N = 281; PP refers to performance pressure; AL refers to authoritarian leadership; ISC refers to independent self-construal; EP refers to employee presenteeism.

**Table 3:** Descriptive statistics and correlation analysis.

Variables	Mean	SD	1	2	3	4	5	6	7	8	9
1 Sex	1.660	0.474	1								
2 Age	32.18	5.663	0.059	1							
3 Education	3.080	0.487	0.100	−0.096	1						
4 Work Time	8.138	4.8602	0.003	0.882 ***	−0.161 **	1					
5 Organization Type	2.860	0.606	−0.035	0.012	0.109	−0.038	1				
6 Performance Pressure	3.202	1.063	0.033	−0.011	−0.031	−0.018	0.004	1			
7 Authoritarian Leadership	2.641	1.053	0.005	−0.065	−0.026	−0.018	0.029	0.287 ***	1		
8 Independent Self-construal	4.054	0.627	0.066	0.062	0.132 *	0.019	0.052	−0.093	−0.065	1	
9 Employee Presenteeism	2.948	1.192	−0.006	−0.120 *	0.029	−0.099	−0.052	0.501 ***	0.350 ***	−0.117 *	1

Note: N = 281; * *p* < 0.05, ** *p* < 0.01, *** *p* < 0.001.

**Table 4:** Regression analysis results.

Variable	Employee Presenteeism						
	Model 1	Model 2	Model 3	Model 4	Model 5	Model 6	Model7
Sex	−0.161	−0.148	−0.174	−0.136	−0.131	−0.122	−0.130
Age	−0.028	−0.031	−0.019	−0.014	−0.013	−0.019	−0.021
Education	0.079	0.122	0.131	0.164	0.178	0.168	0.150
Work Time	0.004	0.011	−0.001	−0.003	−0.004	0.001	0.005
Organization Type	−0.112	−0.116	−0.135	−0.147	−0.143	−0.139	−0.132
PP		0.563 ***	0.490 ***	0.504 ***	0.499 ***	0.494 ***	0.475 ***
AL			0.254 ***	0.248 ***	0.246 ***	0.213 ***	0.23 ***
ISC					−0.098	0.020	−0.054
PP×AL				0.114 *	0.111 *	0.116 *	0.094
PP×ISC							0.097
AL×ISC							−0.173
PP×AL×ISC						−0.248 **	−0.186 *
*F*	1.233	17.136 ***	18.203 ***	16.671 ***	14.937 ***	14.818 ***	12.560 ***
*R^2^*	0.004	0.257	0.301	0.309	0.310	0.330	0.331
Δ*R^2^*	0.022	0.251	0.318	0.011	0.332	0.023	0.360

Note: N = 281; * *p* < 0.05, ** *p* < 0.01, *** *p* < 0.001; PP refers to performance pressure; AL refers to authoritarian leadership; ISC refers to independent self-construal.

**Table 5:** Simple slope analysis 1.

Authoritarian Leadership	Effect	Boot SE	*t*	*p*	Bootstrap 95%CI	
Low levels of AL (M + SD)	0.385	0.077	5.011	0.000	0.234	0.537
High levels of AL (M − SD)	0.660	0.100	6.606	0.000	0.463	0.857

Note: N = 281; AL refers to authoritarian leadership.

**Table 6:** Simple slope analysis 2.

Authoritarian Leadership	Independent Self-Construal	Effect	Boot SE	*t*	*p*	Boot 95% CI	
Low levels of AL	Low levels of ISC	0.167	0.112	1.489	0.138	−0.054	0.389
Low levels of AL	High levels of ISC	0.507	0.085	5.942	0.000	0.303	0.614
High levels of AL	Low levels of ISC	0.715	0.190	3.768	0.000	0.341	1.088
High levels of AL	High levels of ISC	0.533	0.125	4.264	0.000	0.287	0.780

**Table 7:** Hypothesis test results.

Hypothesis	Accept Status
H1: Performance pressure exerts a significant positive effect on employee presenteeism.	Accept
H2: Authoritarian leadership plays an enhancing moderating role in the relationship between performance pressure and employee presenteeism.	Accept
H3: Independent self-construal weakens the enhancing moderating effect of authoritarian leadership on performance pressure and employee presenteeism.	Accept

## Data Availability

The raw data supporting the conclusions of this article will be made available by the authors.

## Appendix A

**Survey Items**
**Performance Pressure:** PP1. I would characterize my workplace as a results-driven environment. PP2. The pressures for performance in my workplace are high. PP3. I feel tremendous pressure to produce results. PP4. If I don’t produce at high levels, my job will be at risk.
**Independent Self-construal:** ISC1. My personal identity, independent of others, is very important to me. ISC2. I enjoy being unique and different from others in many respects. ISC3. I am comfortable with being singled out for praise or rewards. ISC4. Having a lively imagination is important to me. ISC5. I’d rather say “No” directly, than risk being misunderstood.ISC6. Speaking up during a class is not a problem for me.
**Authoritarian Leadership:** AL1. My leader asks me to obey his/her instructions completely. AL2. My leader determined all decisions in the organization whether they are important or not. AL3. My leaders typically possess crucial information but do not share it. AL4. My leader always behaves in a commanding fashion in front of employees. AL5. We have to follow the leader’s rules to get things done. If not, he/she punishes us severely.
**Presenteeism:** Recall whether you have experienced the following situations in the past 6 months: (1). Although you feel sick, you still force yourself to go to work. (2). Although you have physical symptoms such as headache or backache, you still force yourself to go to work.
**Demographic information:** 1. Please select your Sex. (Man/Woman) 2. Please write your age: ______. 3. Please select your educational background. (High school and below/Associate degree/Bachelor’s degree/Master’s degree and above) 4. Work Time (Year): ______. 5. Please select your organization type. (National Government Agencies/State-owned enterprises/Private enterprise/Foreign enterprise/Other)
